# A New Form of Rubber Wedge

**Published:** 1894-06

**Authors:** William Herbert Rollins

**Affiliations:** Boston


### A NEW FORM OF RUBBER WEDGE.

BY WILLIAM HERBERT ROLLINS, BOSTON.

The chief defect of rubber for separating teeth is the liability of the rubber slipping against the gum and injuring it, as well as making it painful. I overcome this by making the wedge of T-shape. The top of the T is narrow and thin, so as to prevent striking it in eating, but yet it is quite sufficient to prevent movement towards the gum.

The second defect of rubber wedges is that they make the teeth sore, but I find that if they are used in four grades, the thinnest not thicker than thick coffer-dam, the work is done gradually and with the minimum of pain. In my own mouth, at least, there is no other way which I will tolerate.

